# Systemic inflammatory indices comprising monocytes provide a clinical significance for thyroid cancer identification

**DOI:** 10.1038/s41598-025-23765-7

**Published:** 2025-11-14

**Authors:** Lobna Refaat, Marwa S. Eissa, Ghada N. Elnaggar, Maha Mehesen, Mohab S. Eissa, Amr Kamal, Mona S. Abdellateif

**Affiliations:** 1 Clinical pathology department, National cancer institute, Cairo University, Cairo, Egypt; 2 Internal Medicine and Endocrinology Department, Faculty of Medicine, Cairo University, Cairo, Egypt; 3 Endocrinology Department, Faculty of Armed Forces of Medical College (AFMC), Cairo, Egypt; 4 Nuclear Medicine Department, National cancer institute, Cairo University, Cairo, Egypt; 5 Pathology Department, National cancer institute, Cairo University, Cairo, Egypt; 6 Surgical Oncology Department, National cancer institute, Cairo University, Cairo, Egypt; 7 Medical Biochemistry and Molecular Biology, Cancer biology department, National cancer institute, Cairo University, Cairo, Egypt; 8 Medical Biochemistry and Molecular Biology, Galala University, Galala City, Egypt

**Keywords:** Thyroid cancer, Inflammation, NLR, PLR, LMR, SII, AISI, SIRI, Biochemistry, Cancer, Biomarkers, Endocrinology, Medical research, Oncology

## Abstract

Is to assess the diagnostic and prognostic role of different inflammatory indices in patients with benign and malignant thyroid nodules. The neutrophil to lymphocyte ratio (NLR), lymphocyte to monocyte ratio (LMR), platelet to lymphocyte ratio (PLR), derived NLR (dNLR), systemic inflammation index (SII), neutrophil to lymphocyte, platelet ratio (NLPR), systemic inflammation response index (SIRI), and aggregate index of systemic inflammation (AISI) were assessed in150 thyroid cancer (TC) patients, 75 benign nodule patients, compared to 70 healthy controls. There was a significant difference among TC patients and control group regarding the PLR, LMR, SII, NLPR, SIRI, and AISI (p = 0.006, p < 0.001, *p* = 0.043,p < 0.001 p < 0.001, and *p* < 0.001; respectively). LMR and SIRI could efficiently differentiate malignant versus benign thyroid nodules at a cutoff of 5.2 and 0.597; respectively. LMR, PLR, SIRI, and AISI were notably associated with high-risk stratification of TC patients (p = 0.011, p = 0.035, p = 0.036, and *p* = 0.034; respectively). Moreover, PLR was significantly elevated in TC patients with lymph node (LN) metastasis (p = 0.010). The LMR (OR = 0.318, p < 0.001), SIRI (OR = 2.293, p = 0.001), AISI (OR = 2.714, *p* < 0.001), and FT4 (OR = 0.250, p < 0.001) could differentiate TC against non-TC groups. LMR, SIRI, AISI, and FT4 are independent risk factors for TC (p < 0.001, p = 0.030, p = 0.026, and *p* < 0.001; respectively). There was no significant impact of the assessed inflammatory indices on the disease-free survival of the patients. LMR, PLR, SII, NLPR, SIRI, and AISI could be potential supportive markers for TC diagnosis. LMR and SIRI could help in differentiating malignant versus benign thyroid nodules.

## Introduction

Thyroid cancer (TC) is the commonest endocrine malignancy globally. Though it represents nearly 1% of all malignant tumors, its incidence is being increased in recent years^1^. It had been reported by the Global Cancer Observatory survey that TC accounted for 586,000 cancer cases worldwide in 2020^2^. TC is a neoplasm of the glandular thyroid tissue, which varies in its pathological subtypes. It is classified into papillary TC (PTC), follicular (FTC), and Huerthle cell tumours, which were stratified as differentiated TC (DTC). The poorly differentiated TC includes the medullary TC (MTC) and anaplastic TC^3^. It had been reported that the 10 years-survival rate of differentiated TC is about 90%. However, regarding the undifferentiated or poorly differentiated TC, it is around 10% due to increased incidence of treatment resistance^4,5^.

The standard care of therapy for TC is the surgical resection, followed by radioactive iodine (RAI) ablation (iodine-131), with thyroxine therapy in most patients^6^. Meanwhile, those with advanced TC or who were refractory to radioiodine therapy were candidates for chemotherapy, immunotherapy, or kinase inhibitors^7^. However, the clinical outcome of those patients is very poor with unfavourable survival rates^1^.

Indeed, proper diagnosis of TC is an important issue, it is primarily depending upon ultrasonography followed by fine needle aspiration cytopathology (FNAC). Both techniques are complementary to each other’s and should be considered before surgical intervention^8^. However, nearly, 8%-20% of the FNAC samples are insufficient for diagnosis, and repetition is required^8–10^. Moreover, it is an invasive technique, and it cannot be used for the follow-up of the patients^1^. Therefore, searching for other diagnostic or prognostic markers is an essential matter for improving the clinical outcome of TC patients^11^.

Accumulating evidence suggested that inflammation have a great implication in cancer initiation and progression^12^. Inflammation influences the tumor microenvironment through the recruitment of different inflammatory cells including macrophages, monocytes, and granulocytes, which leads to uncontrolled production of inflammatory mediators and cytokines^13,14^. This inflammation is also reflected systemically through altered production of hematopoietic cells with increased secretion of acute phase reactants, inflammatory cytokines, chemokines, and growth factors that affect cancer prognosis and outcome^14^.

The blood cells derived inflammatory indices implying lymphocytes, monocytes, neutrophils, and platelets are now extensively assessed for their diagnostic and prognostic roles in different diseases^15–17^. These cells play pivotal roles in shaping the inflammatory status and the immune reactions of the body, that reflect the outcomes of the patients^17^. Currently, different serum inflammatory indices had been identified to have a prognostic and/or predictive values in variable types of cancers involving hematological, hepatocellular, biliary, urinary, head and neck cancers^18–22^. However, their roles in the diagnosis and progression of the different pathological subtypes of TC is still not fully elucidated^23^.

The aim of the current study is to assess the diagnostic and prognostic role of different systemic inflammatory indices including the neutrophil to lymphocyte ratio (NLR), lymphocyte to monocyte ratio (LMR), platelet to lymphocyte ratio (PLR), derived NLR (dNLR), systemic inflammation index (SII), neutrophil to lymphocyte, platelet ratio (NLPR), systemic inflammation response index (SIRI), and aggregate index of systemic inflammation (AISI) in patients with benign and malignant thyroid nodules. Additionally, the impact of these inflammatory indices on patients’ response to therapy, survival rates, and clinical outcomes was also assessed. This could help finding proper useful markers that assist in TC diagnosis especially versus those with benign nodules.

### Patients and methods

This is a retrospective cohort study including 225 patients with single or multiple thyroid nodules, who attended the National Cancer Institute (NCI), Cairo University during the period between 2019 and 2023. The study involved 150 patients diagnosed and confirmed histo-pathologically with TC (malignant thyroid nodules), and 75 patients diagnosed with benign thyroid nodules, compared to 70 normal healthy control subjects. Both sexes were included with an age more than 18 years old. Patients were excluded in case of pregnancy, previous malignancy, history of chemotherapy or radiotherapy, presence of haematological diseases as bleeding tendency, haemophilia, or thrombocytopenia. All the included individuals were subjected to full clinical examination, laboratory assessment in the form of serum free tetraiodothyronine (T4), free triiodothyronine (T3), thyroid stimulating hormone (TSH), and thyroglobulin. Thyroid U/S and FNAC were performed for all patients for confirming the diagnosis and assignment of the groups. Scintigraphy was done for all hyperthyroid patients. TC patients were classified into low-risk stratification, intermediate-risk, and had high-risk disease according to the American Thyroid Association Risk Stratification System and AJCC/TNM Staging System & 2015 ATA guidelines^24^.

The complete blood cell count (CBC)-derived different inflammation markers were assessed in the thyroid nodule patients and control subjects. The inflammatory indices included NLR (neutrophil/lymphocytes), LMR (lymphocyte/monocyte ratio), PLR (platelet/lymphocyte ratio), dNLR (neutrophils/ (white blood cells − neutrophils)), SII (neutrophils × platelets/lymphocytes), NLPR (neutrophil/(lymphocyte × platelet ratio)), AISI (neutrophils × monocytes × platelets/lymphocytes, and SIRI (neutrophils × monocytes/lymphocytes).

The calculated inflammatory indices were correlated with patients’ clinic-pathological features, response to therapy, and disease-free survival (DFS) of the patients (Table 1).

**Table 1:** Clinical and laboratory features of the assessed patients’ groups.

	Control (70)	Benign (75)	Cancer (150)	P value
Age (years)	43.1±16.9	45.6±14.2	45.5±14.4	0.527
TSH (mIU/L)	1.56 (0.5-8.6)	1.07 (0.01-44)	1.61 (0.01-147)	0.344
FT4 (ng/dL)	1.2 (0.5-2.7)^ab^	1.2 (0.4 -6.9)^a^	1.08 (0.08-2.9)^b^	0.002
FT3 (pg/mL)	3 (1-5.4)	2.3 (0.9-6.3)	2.8 (0.4-38)	0.411
Hb (g/dl)	12.5±2	12.3±1.5	12.7±1.6	0.232
RBCs (106/ul)	4.6±0.7	4.6±0.5	4.8±0.5	0.083
HCT (%)	37.8±6.1	37.5±4.5	38.2±4.1	0.539
MCV (fL)	81.7±8.2	81.6±6.3	80.2±7.1	0.234
MCH (Pg)	27.1±3	28.3±9.3	26.5±3	0.080
Platelets (×10^9^/L)	222.5±67 ^a^	287.7±84 ^b^	281.8±80 ^b^	P<0.001
TLCs (×10^9^/L)	7.1 (3.1-18)	7 (3.7-11.2)	7.02 (2.9-23.6)	0.150
Neutrophils (10^3^/ul)	4.2 (1-5.6)	3.9(1.6-6.9)	3.9 (0.88-18.6)	0.922
Lymphocytes (10^3^/ul)	2.27 (1.3-3.4)	2.52 (1.3-4.3)	2.4 (0.87-6.8)	0.279
Monocytes (10^3^/ul)	0.34 (0.05-1.3) ^a^	0.43 (0.06-0.84)^b^	0.5 (0.2-1.77) ^c^	P<0.001
Basophils (10^3^/ul)	0 (0-2)	0.0 (0-1)	0.02 (0-7)	0.087
Eosinophils (10^3^/ul)	1 (0-4) ^a^	2 (1-4) ^b^	0.16 (0-7) ^c^	P<0.001
Creatinine (mg/dL)	0.7 (0.4-1.2) ^a^	0.8 (0.6-1.3) ^b^	0.8 (0.4-4.3) ^b^	0.016
ALT (u/L)	19 (7-35)	20 (12-34)	17 (8-104)	0.378
AST (u/L)	24 (8-44)	24 (12-39)	20 (8-94)	0.772

ALT: alanine transaminase, AST: aspartate transaminase, FT3: Free Triiodothyronine, FT4: Free Thyroxin, Hb: Haemoglobin concentration, HCT: haematocrit value, MCH: mean corpuscular hemoglobin, MCV: mean corpuscular volume, RBCs: red blood cells, TLC: total leukocyte count, TSH: Thyroid Stimulating Hormone. values with different letters are statistically different”. The P value is significant if <0.05.

### Management of the TC patients

Patients who were confirmed for thyroid malignancy underwent surgery in the form of total thyroidectomy [101 (66%) patients], subtotal thyroidectomy [13 (8.5%) patients], and hemi-thyroidectomy [36 (23.5%) patients]. There were 114/150 Patients with malignant thyroid nodules received radioactive iodine (RAI-131) therapy with the dose adjusted according to the American Thyroid Association Risk Stratification System and AJCC/TNM Staging System & 2015 ATA guidelines^24^. Patients with high-risk stratification (17 patients) received a dose of RAI-131 ranged from 120 mCi in five patients, 150 mCi in 9 patients, and 200 mCi in two patients. While patients with intermediate risk (72 patients), 34 patients received 80 mCi, 22 patients received 120 mCi, and four patients received 150 mCi. Regarding low-risk TC patients, 19 received 30 mCi, and 19 received 80 mCi (Table 2).

**Table 2:** The Clinico-pathological characteristics of thyroid cancer patients.

Patients characteristics		Risk stratification			P value
		Low (n=61)	Intermediate (n=72)	High (n=17)	
Age (years)	(median& range)	42 (31-80)	41 (50-74)	60 (52-74)	0.159
Thyroglobulin	(median& range)	6.9 (0.04-15140)	7 (0.04-2263)	1100 (8.4-19390)	P<0.001
Sex	Male	6 (9.8%)	10 (13.9%)	5 (29.4%)	0.120
	Female	55 (90.2%)	62 (86.1%)	12 (70.6%)	
Grade	low	54 (88.5%)	62 (86.1%)	12 (70.6%)	0.175
	high	7 (11.5%)	10 (13.9%)	5 (29.4%)	
Pathological subtypes	Papillary thyroid carcinoma	48 (78.7%)	60 (83.3%)	10 (58.8%)	0.303
	Follicular carcinoma	7 (11.5%)	8 (11.1%)	5 (29.4%)	
	Medullary carcinoma	3 (4.9%)	4 (5.6%)	1 (5.9%)	
	sarcomatoid carcinoma	1(1.6%)	0 (0.0%)	0 (0.0%)	
	Mixed papillary and anaplastic or follicular carcinoma	2 (3.3%)	0 (0.0%)	1(5.9%)	
Surgical treatment	Total thyroidectomy	37 (60.7%)	53 (73.6%)	11 (64.7%)	0.230
	Subtotal thyroidectomy	8 (13.1%)	5 (6.9%)	0 (0.0%)	
	hemi-thyroidectomy	16 (26.2%)	14 (19.4%)	6 (35.3%)	
Lymph node metastasis	No	61 (100%)	27 (37.5%)	5 (29.4%)	p<0.001
	yes	0 (0.0%)	45 (62.5%)	12 (70.6%)	
Distant Metastasis	No	60 (98.4%)	70 (97.2%)	0 (0.0%)	p<0.001
	yes	1 (1.6%)	2 (2.8%)	17 (100%)	
Radiation dose	30	19 (31.1%)	0 (0.0%)	0 (0.0%)	p<0.001
	80	19 (31.1%)	0 (0.0%)	0 (0.0%)	
	100	0 (0.0%)	34 (47.2%)	0 (0.0%)	
	120	0 (0.0%)	22 (30.6%)	5 (29.4%)	
	150	0 (0.0%)	4 (5.6%)	9 (52.9%)	
	200	0 (0.0%)	0 (0.0%)	2 (11.8%)	
Not receiving RAI		23 (37.7%)	12 (16.7%)	1 (5.9%)	
Nodal recurrence	Yes	1 (1.6%)	16 (22.2%)	3 (17.6%)	
	No	60 (98.4%)	56 (77.8%)	14 (82.3%)	
Death	Yes	0 (0.0%)	2 (2.8%)	0 (0.0%)	0.338
	No	60 (100%)	70 (97.2%)	17 (100%)	

Before receiving RAI131, patient with hemithyroidectomy (36/150) were referred to the surgical department for completion. 24 out 36 were of low-risk disease, one patient was of high-risk stratification, while the remaining 11 patients had intermediate-risk TC. Those patients of intermediate and high-risk disease didn’t receive RAI131 as they lost follow up, then they represented to the thyroid department by recurrence.

After ablative RAI dose, all patients were followed up by routine TSH, thyroglobulin, neck US and CT chest every 3 months in first 2 year, then every 6 months in the next 2 years. Diagnostic 5mCi RAI was done after one year from the ablative dose. During the follow up, if there is elevated TG more than 10 ng/mL, 5mCi RAI-131 was administrated to detect the reason for this elevation. If 5mCi RAI was negative, FDG PET/CT were required to detect the cause of this rise. So, the recurrence can be detected either by 5mCi RAI-131, neck US, CT chest or FDG PET/CT. If all of these were negative, we can give a trial of therapeutic dose of RAI131, and scan was done on the 4th day.

Recurrence was encountered in 20/150 patients. Three patients (3/20) were of high-risk disease, one of them didn’t receive RAI131 as he lost follow up then represented by nodal recurrence after nine months. The other two patients showed nodal recurrence after 7 months and 11 months, respectively. Moreover, there was one patient (1/20) with low-risk disease experienced recurrence after two years. Regarding the remaining 16 intermediate-risk TC patients, (11/20) of them didn’t receive the ablative dose as they lost follow up then they represented by nodal recurrence. While the other 5 patients, one of them had local recurrence and 4 out of 5 had nodal recurrence. The date of recurrence ranging from 11 months to 8 years (Table 2).

### Statistical analysis

Data were analysed using the SPSS package (version 22; SPSS Inc., Chicago, IL, USA). normalization was tested using Shapiro test. Continuous variables were expressed as median and interquartile range (IQR), and comparisons between groups were performed using the Mann-Whitney test or Kruskal-Wallis test. Categorical variables were presented as frequencies and percentages, where the Chi-square test for comparison among groups. Comparisons between groups were performed using the Mann-Whitney test and Chi-square test for numerical and categorical variables, respectively. A receiver operating characteristic (ROC) curve analysis was implemented to assess the diagnostic function of the inflammatory indices for TC patients. Univariate and multivariate regression analyses were performed to detect the association of different variables the TC diagnosis. Disease-free survival (DFS) was calculated from date of primary treatment till the date of relapse or progressive disease. Cox-regression hazard model was used to assess factors associated with disease recurrence. P-value was considered statistically significant if *p* < 0.05, where all tested were two-tailed.

## Results

### Clinical and laboratory features of the assessed patients’ groups

The mean ages of the included individuals were 45.5 ± 14.4 years old for TC patients, 45.6 ± 14.2 years old for patients with benign thyroid nodules, and 43.1 ± 16.9 years old for the healthy controls. The pathological types of the malignant nodules were papillary thyroid carcinoma in 118 (78.7%) patients, follicular carcinoma in 20 (13.3%) patients, medullary carcinoma in 8(5.3%) patients, mixed papillary and anaplastic or follicular carcinoma in 3 (2.0%) patients, and finally sarcomatoid carcinoma in only one (0.7%) patient. There was a significant increase in the free tetraiodothyronine (FT4) in patients with benign thyroid nodules [1.2 (0.4–6.9) ng/dL] compared to cancer patients [1.08 (0.08–2.9) ng/dL, *p* = 0.002]. While there was no significant difference among the assessed patients’ groups regarding the FT3 and TSH (*p* = 0.411, *p* = 0.344; respectively). The platelets (PLTs) count was significantly increased in patients with benign and malignant thyroid nodules [287.7 ± 84 and 281.8 ± 80 (×10^9^/L); respectively], in comparison to the normal control subjects [222.5 ± 67 (×10^9^/L), *p* < 0.001]. There was a significant elevation in the monocyte counts in the malignant and benign thyroid nodule patients [0.5 (0.2–1.77) and 0.43 (0.06–0.84) 10^3^/ul; respectively] compared to the normal controls [0.34 (0.05–1.3) 10^3^/ul, *P* < 0.001]. Also, there was a notable increase in the eosinophil counts in in patients with malignant thyroid nodules, relative to those with benign nodules and normal control group [0.16 (0–7), 2 (1–4), and 1 (0–4) 10^3^/ul, *p* < 0.001; respectively]. Moreover, the creatinine level was significantly increased in the TC patients in comparison to those with benign nodules and control group [0.8 (0.4–4.3), 0.8 (0.6–1.3), and 0.7 (0.4–1.2) mg/dL; respectively, *p* = 0.016]. Otherwise, there were no significant differences observed regarding the other clinical and laboratory data among the assessed patients’ groups (Table 1).

Regarding the TC patients, there were 13.3% (20/150) patients with high grade malignancy. Lymph node (LN) metastasis was encountered in 38.0% (57/150) of the patients, and 13.3% (20/150) showed distant metastasis. Distant metastasis was encountered in 17 patients at the start of the disease, and 3 patients were detected during postoperative follow up. Out of these 17 patients who had distant metastasis, there were four patients who showed nodal recurrence after treatment.

There were 40.7% (61/150) patients classified as low-risk stratification, 48% (72/150) with intermediate-risk, and 11.3% (17/150) had high-risk disease. Nodal recurrence was experiences in 13.3% (20/150) of the patients. Other clinical features of TC patients were presented in Table 2.

### Assessment of the inflammatory indices among the thyroid patients’ groups

The LMR was significantly decreased in TC patients [4.8 (1-14.9)], compared to both the benign [5.8 (1.6–36)], and the control groups [7.1 (1.9–24), *p* < 0.001]. Additionally, it was significantly decreased in the benign nodule patients in relation to the control group (*p* < 0.001). There was a significant increase in the PLR value in TC patients compared to the control group [114 (25–428) versus 91.7 (29.5–245); respectively, *p* = 0.006]. However, there was no significant difference between the benign nodule patients [106 (50–250)], and cancer patients or the control group. Similarly, the SII was notably increased in TC patients compared to the control group [429 (61-7562) versus 363 (75–934); respectively, *p* = 0.043]. However, there was no significant difference between the benign nodule patients [412 (134–1110)], and those with TC or the control group.

Regarding the NLPR, there was a significant decrease in patient with benign or malignant thyroid nodules compared to the control subjects [0.006 (0.002–0.03), 0.006 (0.002–0.04), and 0.008 (0.002–0.05); respectively, *p* < 0.001]. While there was no significant difference in the NLPR between the benign nodule patients and those with TC. The SIRI was significantly increased in TC patients in comparison to the control group or those with benign nodules [0.8 (0.1–8.8), 0.56 (0.11–2.1), and 0.59 (0.11–4.3); respectively, *p* < 0.001]. While there was no significant change between the benign nodule patients and the control group. Moreover, the AISI was notably increased in patient with benign or malignant thyroid nodules compared to the control subjects [150 (30–930), 206 (16-6681), and 117 (15–681); respectively, *p* < 0.001]. However, there was no significant difference in the AISI value between the benign nodule patients and TC patients.

**Fig. 1:**
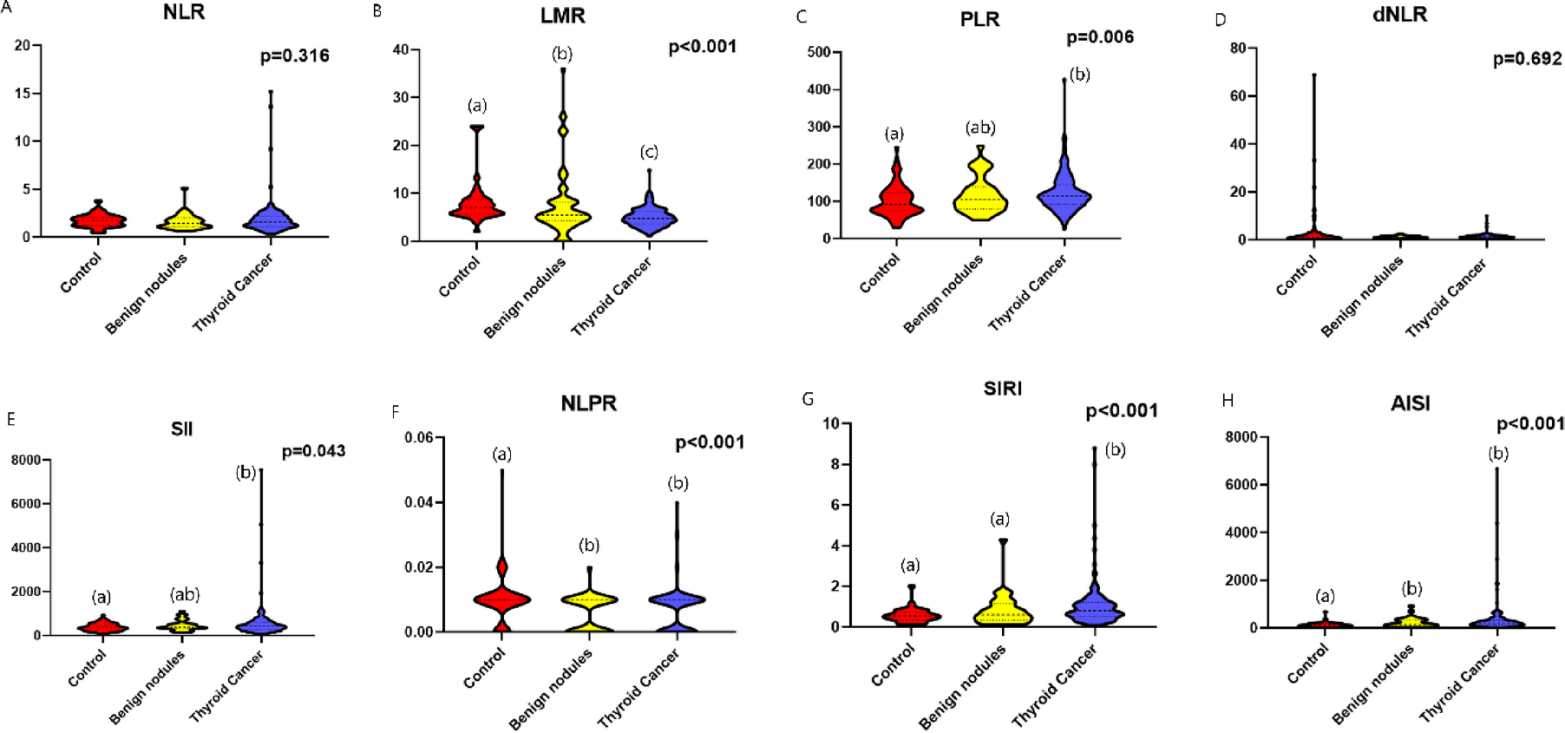
Assessment of different inflammatory indices including; (**A**) NLR, (**B**) LMR, (**C**) PLR, (**D**) dNLR, (**E**) SII, (**F**) NLPR, (**G**) SIRI, and (**H**) AISI among the thyroid patients’ groups. Ns: not significant, * statistically significant.

On the other hand, there was no significant variation in the NLR among the TC patients, benign thyroid nodule patients, and normal control subjects [1.6 (0.34–15.2), 1.8 (0.63–5.14), and 1.8 (0.42–3.8); respectively, *p* = 0.316]. Similarly, there was no significant difference in the dNLR among the TC patients, benign thyroid nodule patients, and normal control subjects [1.2 (0.3–10), 1.2 (0.6–2.6), and 1.3 (0.11-69); respectively, *p* = 0.692, Fig. 1].

### Association of the inflammatory indices with TC patients’ clinical features

Thyroid cancer patients with high-risk stratification showed a significant decrease in the LMR in comparison to those with low or intermediate-risk disease [3.7 (1.6–7.7), 4.7 (1–10), and 5 (2.3–15); respectively, *p* = 0.011]. Similarly, there was a significant increase in the PLR in high-risk group patients relative to patients with low or intermediate-risk disease [122 (44–265), 117 (25–428), and 105 (42–247); respectively, *p* = 0.035]. In addition, SIRI was notably increased in high-risk group patients in comparison to patients with low or intermediate-risk disease [1.1 (0.3-8), 0.76 (0.2–8.8), and 0.73 (0.1–4.4); respectively, *p* = 0.036]. Finally, there was a significant association between high-risk stratification and increased AISI compared to patients with low or intermediate-risk disease [271 (103–2884), 245 (39-6681), and 173 (16-1603); respectively, *p* = 0.034, Table 3]. Moreover, PLR was significantly elevated in TC patients with lymph node (LN) metastasis in relation to those with negative LN metastasis [117 (25–428) versus 107 (42–265); respectively, *p* = 0.010, Table 4]. On the other hand, there was no significant association among the other inflammatory indices and the assessed clinico-pathological features including pathological types (not presented), tumour grade, risk stratification, disease recurrence, distant and LN metastasis (Tables 3, 4).

**Table 3:** Association of the inflammatory indices with the tumor grade and clinical risk stratification.

	Tumor grade		p value	Risk stratification			p value
	Low	High		Low	Intermediate	High	
NLR	1.6 (0.3-15.2)	1.5 (0.4-2.8)	0.853	1.7 (0.4-15)	1.5 (0.4-5.3)	1.8 (0.9-9.2)	0.176
LMR	4.8 (1-14.9)	4.5 (2.1-7.7)	0.597	4.7 (1-10)	5 (2.3-15)	3.7 (1.6-7.7)	**0.011**
PLR	114 (25-428)	103 (53-177)	0.538	117 (25-428)	105 (42-247)	122 (44-265)	**0.035**
dNLR	1.2 (0.3-10)	1.2 (0.3-2.6)	0.883	1.3 (0.3-10)	1.1 (0.3-3)	1.2 (0.7-5)	0.297
SII	437 (61-6562)	406 (72-1197)	0.935	474 (72-7562)	363 (61-1931)	399 (220-3315)	0.054
NLPR	0.006 (0-0.04)	0.007 (0-0.01)	0.883	0.006 (0-0.04)	0.006 (0-0.02)	0.008 (0-0.03)	0.275
SIRI	0.8 (0.1-18)	1 (0.2-3.8)	0.623	0.76 (0.2-8.8)	0.73 (0.1-4.4)	1.1 (0.3-8)	**0.036**
AISI	206 (16-668)	233 (39-1832)	0.793	245 (39-6681)	173 (16-1603)	271 (103-2884)	**0.034**

Significant values are in bold.

**Table 4:** Association of the inflammatory indices with tumor metastasis and disease recurrence.

Test variables		LNs metastasis	p value	Distant metastasis	p value	Recurrence	p value
NLR	-ve	1.6 (0.4-15)	0.461	1.6 (0.3-15)	0.670	1 .6 (0.3-15)	0.817
	+ve	1.5 (0.3-9.2)		1.6 (0.9-9.2)		1.5 (0.6-2.5)	
LMR	-ve	4.9 (1-10)	0.924	4.9 (1-15)	0.097	4.9 (1-15)	0.799
	+ve	4.7 (1.6-15)		4.2 (1.6-10)		4.6 (2.6-10)	
PLR	-ve	107 (42-265)	0.010	113 (25-428)	0.190	114 (25-428)	0.918
	+ve	117 (25-428)		122.9 (44-265)		110 (68-221)	
dNLR	-ve	1.2 (0.3-10)	0.406	1.2 (0.3-10)	0.987	1.2 (0.3-10)	0.696
	+ve	1.2 (0.3-5)		1.2 (0.7-5.4)		1.1 (0.5-1.9)	
SII	-ve	453 (72-7562)	0.053	437 (61-7562)	0.715	423 (61-7562)	0.595
	+ve	367 (61-3315)		399 (220-3315)		447 (106-985)	
NLPR	-ve	0.001 (0-0.04)	0.589	0.006 (0-0.04)	0.459	0.006 (0-0.04)	0.346
	+ve	0.006 (0-0.03)		0.007 (0-0.03)		0.005 (0-0.01)	
SIRI	-ve	0.8 (0.2-18)	0.821	0.77 (0.1-18)	0.235	0.8 (0.1-8.8)	0.658
	+ve	0.8 (0.1-8)		1.1 (0.2-8)		0.9 (0.2-2)	
AISI	-ve	238 (39-6681)	0.231	206 (16-6681)	0.439	204 (16-6681)	0.380
	+ve	190 (16-2884)		216 (72-17.7)		249 (37-650)	

### Univariate and Multivariate analysis for the diagnosis of TC patients versus patients with benign nodules and control subjects

Univariate and Multivariate logistic regression analysis was performed to assess the potential of the assessed inflammatory indices, TG, TSH, FT3, and FT4 for the detection of TC patients (150 patients) against those with benign nodules and control subjects together (145 individuals). The univariate analysis revealed that LMR (odds ratio (OR) = 0.318, *p* < 0.001), SIRI (OR = 2.293, *p* = 0.001), AISI (OR = 2.714, *p* < 0.001), and FT4 (OR = 0.250, *p* < 0.001) could significantly differentiate TC against benign nodules patients and control subjects. Additionally, the multivariate analysis showed that LMR, SIRI, AISI, and FT4 are independent risk factors for association with TC (*p* < 0.001, p = 0.030, *p* = 0.026, and *p* < 0.001; respectively, Table 5).

**Table 5:** Univariate and Multivariate analysis for the diagnosis of TC patients versus non-TC patients.

	OR	95% C.I. for EXP(B)		P value
		Lower	Upper	
Univariate analysis				
NLR (<1.6 vs >1.6)	0.930	0.585	1.478	0.757
LMR (<4.8 vs >4.8)	0.318	0.192	0.525	**P<0.001**
PLR (<114 vs >114)	1.488	0.929	2.385	0.099
dNLR (<1.2 vs >1.2)	1.040	0.654	1.652	0.870
SII (<429 vs >429)	1.444	0.901	2.316	0.127
NLPR (<0.006 vs >0.006)	0.695	0.434	1.114	0.131
SIRI (<0.8 vs >0.8)	2.293	1.409	3.731	**0.001**
A ISI (<206 vs >206)	2.714	1.642	4.487	**P<0.001**
TSH (<1.61 vs >1.61)	1.449	0.914	2.300	0.115
FT4 (<1.08 vs >1.08)	0.250	0.144	0.433	**P<0.001**
FT3 (<2.8 vs >2.8)	0.772	0.459	1.299	0.330
Multivariate analysis				
LMR (<4.8 vs >4.8)	0.188	0.083	0.426	**P<0.001**
SIRI (<0.8 vs >0.8)	0.308	0.107	0.890	**0.030**
AISI (<206 vs >206)	2.894	1.134	7.384	**0.026**
FT4 (<1.08 vs >1.08)	0.246	0.134	0.449	**P<0.001**

### The diagnostic potential of the inflammatory indices in TC patients against normal control

**Fig. 2:**
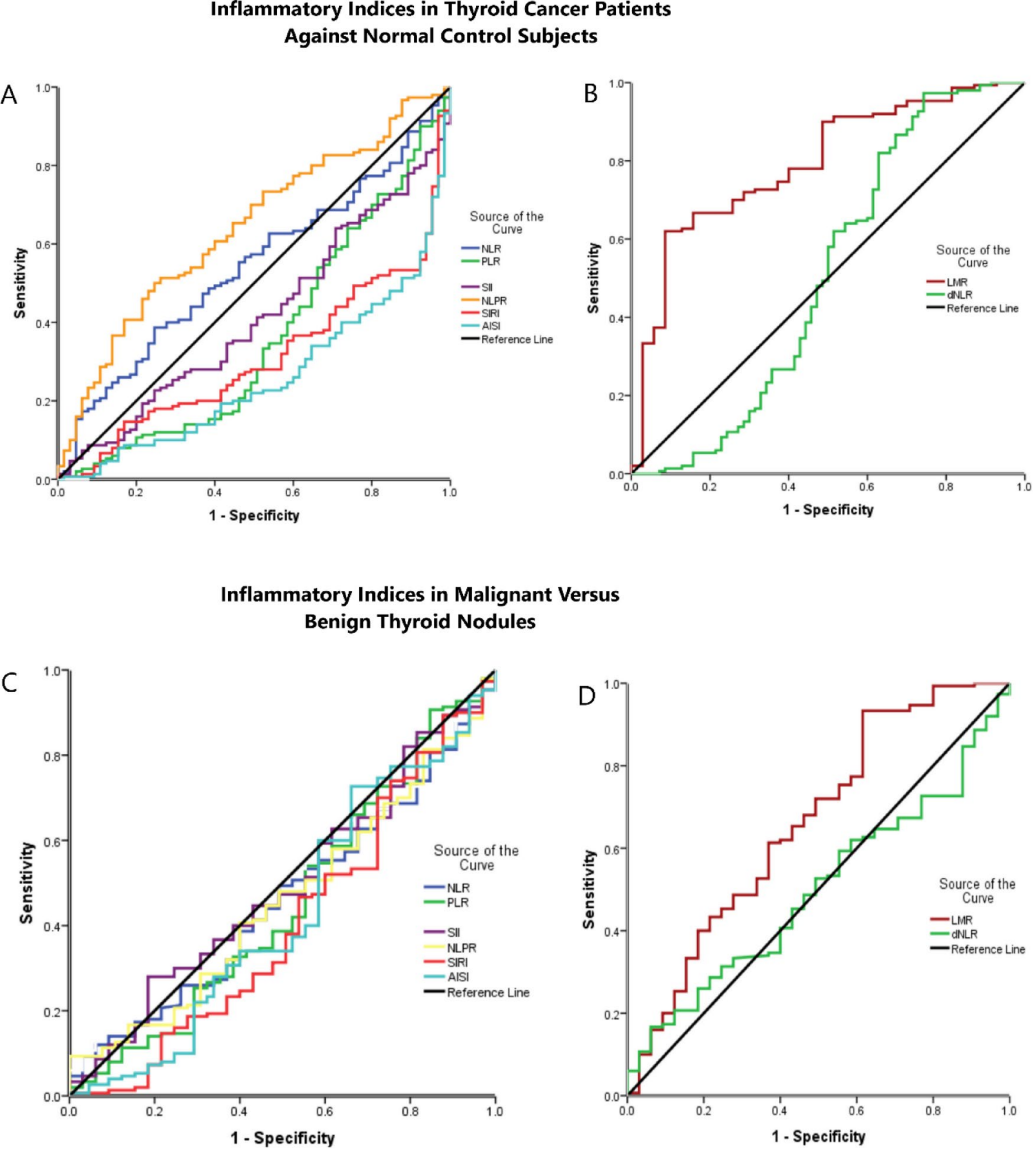
ROC curve analysis of (**A**) NLR, PLR, SII, NLPR, SIRI, AISI, and (**B**) LMR, dNLR for differentiating TC patients against normal control subjects. (**C**) ROC curve analysis of NLR, PLR, SII, NLPR, SIRI, AISI, and (**D**) LMR, dNLR for differentiating patients with malignant thyroid nodules versus those with benign nodules.

ROC curve analysis was performed to differentiate the TC patients against normal control subjects. It showed that the AUC, sensitivity, and specificity of LMR were [0.792, 72.7%, 68.6%; respectively, *p* < 0.001] at a cutoff of 5.97. While those of PLR were [0.638, 60%, and 60%; respectively, *p* = 0.001] at a cutoff of 107. Similarly, the SII achieved a sensitivity of 72%, specificity of 41.5%, AUC of 0.587, at a cutoff of 322.5, (p = 0.043). The NLPR showed an AUC of 0.360, a sensitivity of 41.3%, a specificity of 40%, with a cutoff of 0.007, (*p* = 0.001). The SIRI had a 0.690 AUC, 72% sensitivity, 56.9% specificity, with 0.57 cutoff value, (*p* < 0.001). The AISI showed an AUC of 0.742, a sensitivity of 75.3%, a specificity of 60%, cutoff value of 132.3, (*p* < 0.001). However, there was no significant potential of NLR and dNLR for the diagnosis of TC, where the AUC, cutoff value, sensitivity, and specificity of NLR were [0.460, 1.56, 51.3%, and 40%; respectively, *p* = 0.353], and those of dNLR were [0.467, 1.03, 65.3%, and 41.5%; respectively, *p* = 0.448, Table 6; Fig. 2A, B].

**Table 6:** ROC curve analysis for inflammatory indices in thyroid cancer patients against normal control and benign thyroid nodules.

Variables	TC against normal control					Malignant versus benign thyroid nodules				
	AUC	cutoff	Sensitivity	Specificity	P value	AUC	Cutoff	Sensitivity	Specificity	P value
NLR	0.460	1.56	51.3%	40%	0.353	0.462	1.59	50.7%	52.3%	0.376
LMR	0.792	5.97	72.7%	68.6%	**P<0.001**	0.661	5.2	61.3%	57.7%	**0.012**
PLR	0.638	107	60%	60%	**0.001**	0.453	105.8%	61.3%	52.3%	0.275
dNLR	0.467	1.03	65.3%	41.5%	0.448	0.498	1.03	65.3%	40%	0.968
SII	0.587	322.5	72%	41.5%	**0.043**	0.492	366.5	60%	43.1%	0.845
NLPR	0.360	0.007	41.3%	40%	**0.001**	0.460	0.004	68%	40%	0.347
SIRI	0.690	0.57	72%	56.9%	**P<0.001**	0.404	0.597	68.7%	50.8%	**0.026**
AISI	0.742	132.3	75.3%	60%	**P<0.001**	0.428	150.4	66%	52.3%	0.096

Significant values are in bold.

### The diagnostic potential of the inflammatory indices in malignant versus benign thyroid nodules

The ROC analysis was also performed to detect patients with malignant thyroid nodules versus those with benign nodules. It showed that LMR can significantly differentiate TC patients against those with benign nodules at a cutoff of 5.2, an AUC of 0.605, 61.3% sensitivity, and 57.7% specificity, (*p* = 0.012). Similarly, SIRI can differentiate between patients with malignant thyroid nodules and those with benign nodules at a cutoff of 0.597, an AUC of 0.596, 68.7% sensitivity, and 50.8% specificity, (*p* = 0.026). While the other assessed indices including NLR, PLR, dNLR, SII, NLPR, and AISI had no significant implication for diagnosing malignant versus benign thyroid nodules, as shown in Table 5, (Fig. 2C, D).

### Disease-Free survival of TC patients

There were 20/150 (13.3%) patients experienced nodal recurrence after surgery. The median DFS time of the assessed TC patients was 104.2 months (range: 4.7-108.5 months), with six patients showed recurrence in the first year. 90% (18/20) had papillary carcinoma with 15% (3/20) had high grade disease. LN metastasis was reported in 90% (18/20) of them, and distant metastasis was found in 20% (4/20) of the patients. Intermediate risk was detected in 80% (16/20), and high-risk disease was detected in 15% (3/20) patients. Fifteen of them (75%) received I-131 therapy, and finally, death was encountered in two of them (10%).

**Fig. 3:**
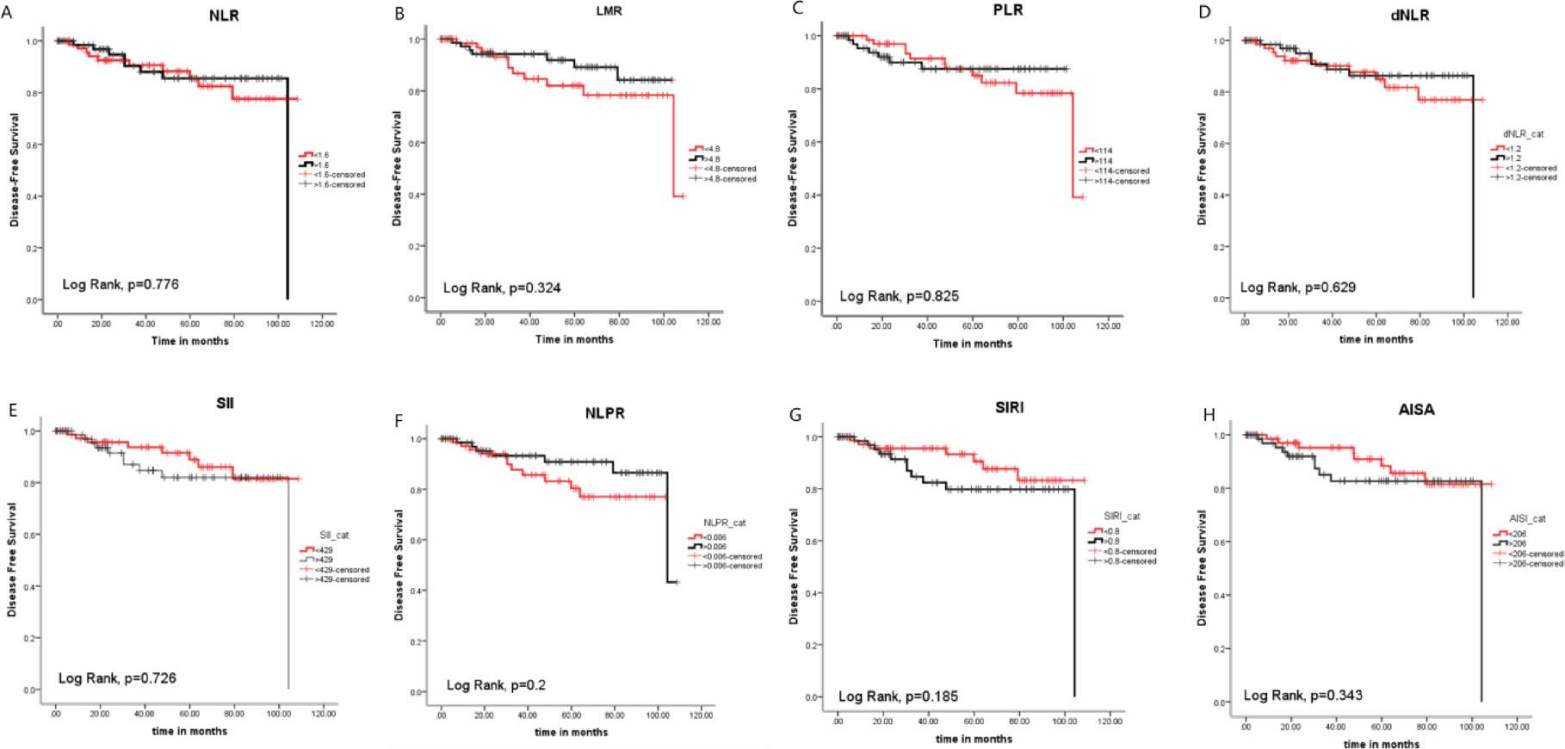
Impact of (**A**) NLR, (**B**) LMR, (**C**) PLR, (**D**) dNLR, (**E**) SII, (**F**) NLPR, (**G**) SIRI, and (**H**) AISI on the DFS rate of TC patients.

Survival analysis showed that there was no significant impact of the assessed inflammatory indices on the DFS of the patients (Fig. 3). Cox regression hazards model showed that LN metastasis associated significantly with nodal recurrence in TC patients. However, there was no significant association between the inflammatory indices, TSH, FT4, FT3, and TG with the DFS of the TC patients (Table 7). On the other hand, SIRI showed a significant impact on sub-stratifying recurrence in patients received I-131 therapy [HR = 8.991, *p* = 0.031, CI = 1.219–66.302]. Patients who showed nodal recurrence after I-131 therapy were 15/114 (13.15%). Out of theses 15 patients who experienced nodal recurrence with I-131 therapy, there were 10 (66.7%) patients had SIRI > 0.8. Therefore, TC patients with elevated SIRI had higher recurrence rates compared to those without elevated SIRI levels (Supp. 1).

**Table 7:** COX regression hazard ratio for the DFS of the TC patients.

	HR	95.0% CI for Exp(B)		P value
		Lower	Upper	
Age (<45.5 vs >45.5)	0.510	0.182	1.431	0.201
Sex (male vs female)	1.165	0.265	5.119	0.840
Grade (low vs high)	0.989	0.276	3.542	0.986
Pathology (papillary vs non-papillary)	0.615	0.140	2.695	0.519
Risk (high vs low & intermediate)	0.485	1.560	0.448	5.436
LN metastasis (+ve vs -ve)	12.096	2.779	52.648	0.001
Distant metastasis (+ve vs -ve)	2.074	0.675	6.372	0.203
I-131(+ve vs -ve)	0.639	0.173	2.352	0.5
NLR (<1.6 vs >1.6)	1.315	0.500	3.461	0.579
LMR (<4.8 vs >4.8)	0.677	0.254	1.801	0.434
PLR (<114 vs >114)	0.865	0.329	2.278	0.770
dNLR (<1.2 vs >1.2)	0.693	0.264	1.822	0.457
SII (<429 vs >429)	1.371	0.528	3.558	0.517
NLPR (<0.006 vs >0.006)	0.474	0.171	1.312	0.151
SIRI (<0.8 vs >0.8)	1.616	0.611	4.273	0.334
AISI (<206 vs >206)	1.773	0.673	4.667	0.246
TSH (<1.61 vs >1.61)	0.795	0.303	2.090	0.642
FT4 (<1.08 vs >1.08)	1.898	0.666	5.410	0.231
FT3 (<2.8 vs >2.8)	1.748	0.584	4.503	0.284
TG (<8.9 vs >8.9)	1.027	0.380	2.778	0.685

## Discussion

Inflammation is an important component of tumour microenvironment. It plays a fundamental role in cancer pathogenesis, progression and invasion^25^. The inflammatory mediators together with the inflammatory cells produced in the tumour microenvironment have a critical impact on the prognosis, aggressiveness, and outcomes of the patients^26^. These key inflammatory cells including lymphocytes, monocytes, neutrophils, macrophage, and platelets produced many inflammatory mediators and cytokines e.g. tumor necrosis factor-α (TNF-α), tumor growth factor-β (TGF-β), interleukin-6 (IL6), and IL1. In the last few years, different inflammatory indices had been developed depending upon the interplay of the inflammatory reaction and the host immune response. These systemic inflammatory indices showed great success in the diagnosis and/or the prognosis of many cancers and different diseases^18–23,27^. Unlike other diagnostic modalities, assessing blood cell-derived inflammatory indices is a rapid, easy, nonpainful, and non-invasive method that can represent the clinical features of the patients. The current study showed that PLR, SII, SIRI, AISI, and NLPR were notably increased in TC patients compared to the control group. In agreement with these data, An Italian study performed by Kars and his colleagues reported a significant increase of SII in DTC patients compared to the control subjects^28^. They also found that SII could differentiate TC patients with a sensitivity of 72.7% and a specificity of 67.9% at a cut-off point of 454.5 × 10^9^ cells/L^28^. Comparably, the present study showed that SII had a 72% sensitivity and 41.5% specificity for the detection of TC patients at a cut-off of 322.5 × 10^9^ cells/L. Ozmen et al. concluded that PLR is a useful marker for the diagnosis of DTC compared to C-reactive protein^29^. Similarly, Offi et al.^30^, observed a significant increase in the PLR value in TC patients, however this significance was weak indicative by a non-significant OR. Additionally, Cao et al., demonstrated a significant association of SII and AISI with the incidence of thyroid nodules in patients with diabetes mellites type II^31^.

Furthermore, the present data showed that LMR was significantly decreased in TC patients, compared to both the benign, and the control groups. Also, it was significantly decreased in the benign nodule patients in relation to the control group. LMR could significantly differentiate TC patients against control subjects with a sensitivity of 72.7% and a specificity of 68.6% at a cutoff lower than 5.97. While it can differentiate patients with malignant thyroid nodules versus those with benign nodules with a 61.3% sensitivity and a 57.7% specificity at a cutoff lower than 5.2. In line with these results, Offi et al. demonstrated that baseline LMR value ≥ 4.09 was a marker for benign nodules, and a value < 4.09 was indicative of malignancy^30^.

Though NLR was reported in several research as a possible diagnostic or prognostic marker in different types of TC^27,29,30,32^. The current study could not detect any significant variation in the NLR and the dNLR among the TC patients, benign thyroid nodule patients, and normal control subjects. This is consistent with that reported by Offi et al., who found no significant difference in NLR values among thyroid nodule patients and control subjects^30^. Similarly, a meta-analysis performed by Liu et al., revealed that NLR did not significantly differ between TC patients and those with benign nodules^33^.

Regarding the association of the inflammatory indices with the disease prognosis and patients’ outcome. The present data showed that decreased LMR value, increased PLR, SIRI, and AISI were notably associated with high-risk stratification of TC patients compared to those with low or intermediate-risk disease. Moreover, PLR was significantly elevated in TC patients with LNs metastasis in relation to those with negative LN metastasis. While the other inflammatory indices showed no significant association with the clinico-pathological features of the patients. These observations are consistent with that reported in literature that low LMR values were accompanied with poor prognosis and inferior outcomes of TC patients^34,35^. Likewise, many series found that PLR value increased significantly with LN metastasis in TC patients^27,36,37^. Also, many studies concluded that elevated PLR value associated with poor prognosis of TC patients^27,30,32^. On the contrary, research conducted by Figueiredo et al., stated that NLR, PLR, and SII associated significantly with aggressiveness of medullary TC, but there was no substantial impact on LN affection or distant metastasis^38^. Additionally, Pang et al., found a notable association of NLR, MLR, SIRI, and AISI with central LN metastasis in PTC patients, while PLR did not show a significant relation^39^.

The current data showed that increased SII was found in TC patients with LN metastasis, however, the value is near significance in the assessed cohort (*p* = 0.053). This finding is comparable to that reported in many articles that SII associated significantly LN affection in TC^40–42^. However, other series proposed that SII had no impact on the histological type, capsule invasion, LN or extrathyroidal extension in DTC^28,37^.

In agreement with many reports in the literature, the current findings demonstrated that NLR did not have a significant impact on the TC aggressiveness in the form of tumor grade, stage, recurrence, distant or LN metastasis^42–44^. However, others reported a notable association of NLR with inferior prognosis of TC patients^38,39^.

Furthermore, the present data demonstrated that there was no significant impact of the assessed inflammatory indices on the disease recurrence or DFS of the patients. Additionally, the Cox regression hazards model showed no significant association between the assessed inflammatory indices, TSH, FT4, FT3, and TG on the DFS of the TC patients except for LN affection. In consistent with these findings, several studies proposed that there was no significant association between NLR^34,45^, LMR^30^, PLR^30,34^ and the DFS rates of TC patients. On the other side, many series concluded a substantial association of NLR with low DFS in TC patients^37,46^. Offi et al. demonstrated that NLR associated with the risk of TC recurrence only when adjusted for age^30^. The current data showed also that SIRI has a significant impact on sub-stratifying recurrence in patients received I-131 therapy. In other words, TC patients receiving I-131 therapy with elevated SIRI > 0.8 had higher recurrence rates compared to those without elevated SIRI levels. These data could direct attentions for the pretreatment assessment of SIRI as an important systemic indicator of potential disease recurrence. In line with these results, Wang et al., demonstrated that SIRI had a notable impact on the response to radioactive iodine therapy in DTC patients at optimal cutoff of 0.47^15^. Therefore, SIRI could be a valuable predicting index for response to I-131 therapy and early nodal recurrence in TC patients. This will allow for better management of the patients with high risk of TC recurrence, if SIRI has been taken into consideration for follow-up of these patients to avoid unsatisfactory I-131 therapy. Potentially, if systematic inflammation causes I-131 failure, there might be a role for pretreatment of anti-inflammatory medications before I-131 therapy.

Indeed, there were about 70% of thyroid surgeries are unnecessary, especially in Tir3a nodules^8,30^. Therefore, the identification of useful markers that can support the differentiation of malignant thyroid nodules is of great importance for the clinical set. The current research provides evidence that systemic inflammatory indices including LMR, PLR, SII, NLPR, SIRI, and AISI could significantly assist the diagnosis of TC patients against normal controls with a sensitivity reaching up to 75.3% for AISI, and a specificity of 68.6% for LMR. Moreover, LMR and SIRI could efficiently help in the differentiation of malignant versus benign thyroid nodules at a cutoff of 5.2 and 0.597; respectively. The presented data were also supported by a multivariate analysis which revealed that LMR, SIRI, AISI, and FT4 could be considered independent risk factors for TC development. Therefore, LMR, SIRI, and AISI are more superior for TC diagnosis than other inflammatory indices including NLR, PLR, dNLR, NLPR, and SII. These inflammatory indices reflect the interplay of many important immune cells and inflammatory cells comprising the neutrophils, monocytes, lymphocytes, and platelets. Neutrophils had been reported to be elevated in TC that associated substantially with poor patients’ prognosis^47^. Increased neutrophils in cancer may be a part of chronic inflammation induced by tissue destruction and cytokine release, or as a part of a paraneoplastic syndrome^48^. On the other side, lymphocytes infiltration is a good indicator of cytotoxic cell death and cancer cell inhibition^49^. Therefore, low lymphocyte counts are linked with immune system suppression and poor patients’ prognosis^50^. While Platelets are reported to surround the tumor cells, thus increasing their adhesion, and preventing cancer cell death. They also secrete some mediators, including TGF-β, platelet-derived growth factor (PDGF), and vascular endothelial growth factor (VEGF), which lead to cancer promotion, progression, and metastasis^51^.

Additionally, these findings highlighted the importance of monocyte, which is a component of both LMR, SIRI, and AISI. Monocytes are a chief regulator of cancer development and metastasis^52^. Moreover, they are the primary origin for dendritic cells and macrophages which have a pivotal role in formulating the tumor microenvironment^53^. Thus, paying attention to this type of cell could provide new insight into TC diagnosis and management. Enhance research are required for the utility of these cells as a therapeutic target for TC patients.

In conclusion, Systemic inflammatory indices including LMR, PLR, SII, NLPR, SIRI, and AISI could be used as potential accurate, accessible, nonpainful, non-invasive, and cost-effective markers that support TC diagnosis. Thyroid cancer patients with high-risk stratification showed a substantial decrease in the LMR value, and an increased PLR, SIRI, or AISI, compared to those with low or intermediate-risk disease. Moreover, PLR was significantly associated with LN metastasis in TC patients. Multivariate analysis revealed that LMR, SIRI, AISI, and FT4 are independent risk factors for TC development. Another important finding is that LMR [AUC of 0.605, 61.3% sensitivity, and 57.7% specificity at a cutoff of 5.2] and SIRI [AUC of 0.596, 68.7% sensitivity, and 50.8% specificity at a cutoff of 0.597] could help in differentiating patients with malignant versus benign thyroid nodules, that will support the FNAC and sonography, especially in patients with intermediate nodules. Moreover, SIRI could be a valuable indicator for response to I-131 therapy and disease recurrence. Consequently, there is a growing body of evidence that the systemic inflammatory indices have an important impact on TC patients’ prognosis and outcomes, that could direct physicians towards the proper line of treatment. Therefore, further research is required to make use of these indices as potential therapeutic targets for TC patients.

The limitation of the work is that; it is based on a relatively small sample size of patients, and it is conducted at a single center with a retrospective design. Therefore, it is recommended to validate these results in a prospective study with a larger scale of patients from different medical centers. This could help for the generalizability of the results.

## Supplementary Information

Below is the link to the electronic supplementary material.

Supplementary Material 1

## Data Availability

The datasets used and/or analysed during the current study are available from the corresponding author on reasonable request.
