# Supplementary material for: Systemic inflammatory indices comprising monocytes provide a clinical significance for thyroid cancer identification
Source: Sci Rep. 2025 Nov 14;15:39967. doi: 10.1038/s41598-025-23765-7 (PMC12618645; doi:10.1038/s41598-025-23765-7)
Supplement: Supplementary file 1 — Supplementary Material 1 [file 41598_2025_23765_MOESM1_ESM.docx]

**Supp. 1**: Role of inflammatory indices in sub stratifying recurrence in patients according to I-131 therapy

| **Indices** | **HR** | **95.0% CI for Exp(B)** | | **P value** |
| --- | --- | --- | --- | --- |
|  |  | **Lower** | **Upper** |  |
| NLR (<1.6 vs >1.6) | 2.122 | 0.010 | 444.916 | 0.783 |
| LMR (<4.8 vs >4.8) | 0.666 | 0.178 | 2.485 | 0.545 |
| PLR (<114 vs >114) | 0.691 | 0.245 | 1.947 | 0.484 |
| dNLR (<1.2 vs >1.2) | 0.153 | 0.001 | 32.426 | 0.492 |
| SII (<429 vs >429) | 2.426 | 0.529 | 11.130 | 0.254 |
| NLPR (<0.006 vs >0.006) | 0.349 | 0.099 | 1.226 | 0.100 |
| SIRI (<0.8 vs >0.8) | 8.991 | 1.219 | 66.302 | **0.031** |
| AISI (<206 vs >206) | 0.341 | 0.077 | 1.509 | 0.156 |
